# Diagnostic Accuracy of a New Cardiac Electrical Biomarker for Detection of Electrocardiogram Changes Suggestive of Acute Myocardial Ischemic Injury

**DOI:** 10.1111/anec.12109

**Published:** 2013-10-07

**Authors:** David M. Schreck, Robert D. Fishberg

**Affiliations:** ^1^ Departments of Emergency Medicine and Internal Medicine Summit Medical Group Berkeley Heights NJ; ^2^ Department of Medicine, Atlantic Health System Overlook Medical Center Summit NJ; ^3^ Department of Medicine and Division of Cardiology, Atlantic Health System Overlook Medical Center Summit NJ

**Keywords:** electrocardiography, acute myocardial infarction, acute myocardial ischemia, myocardial injury, biomarker, chest pain

## Abstract

**Objective:**

A new cardiac “electrical” biomarker (CEB) for detection of 12‐lead electrocardiogram (ECG) changes indicative of acute myocardial ischemic injury has been identified. Objective was to test CEB diagnostic accuracy.

**Methods:**

This is a blinded, observational retrospective case‐control, noninferiority study. A total of 508 ECGs obtained from archived digital databases were interpreted by cardiologist and emergency physician (EP) blinded reference standards for presence of acute myocardial ischemic injury. CEB was constructed from three ECG cardiac monitoring leads using nonlinear modeling. Comparative active controls included ST voltage changes (J‐point, ST area under curve) and a computerized ECG interpretive algorithm (ECGI). Training set of 141 ECGs identified CEB cutoffs by receiver‐operating‐characteristic (ROC) analysis. Test set of 367 ECGs was analyzed for validation. Poor‐quality ECGs were excluded. Sensitivity, specificity, and negative and positive predictive values were calculated with 95% confidence intervals. Adjudication was performed by consensus.

**Results:**

CEB demonstrated noninferiority to all active controls by hypothesis testing. CEB adjudication demonstrated 85.3–94.4% sensitivity, 92.5–93.0% specificity, 93.8–98.6% negative predictive value, and 74.6–83.5% positive predictive value. CEB was superior against all active controls in EP analysis, and against ST area under curve and ECGI by cardiologist.

**Conclusion:**

CEB detects acute myocardial ischemic injury with high diagnostic accuracy. CEB is instantly constructed from three ECG leads on the cardiac monitor and displayed instantly allowing immediate cost‐effective identification of patients with acute ischemic injury during cardiac rhythm monitoring.

It has been reported that coronary heart disease leading to acute coronary syndromes (ACS) is still the number one cause of mortality in the United States,^1^ and chest pain accounts for more than 8 million emergency department (ED) visits annually. 2 It has also been reported hat missed diagnoses of acute myocardial infarction (AMI) is among the highest causes of litigation in the ED^3^, ^4^ and that 2.1% of patients with AMI are discharged from the ED without recognition.^5^

The 12‐lead electrocardiogram (ECG) is the first and single most important test in the initial evaluation of chest pain patients presenting to the ED^6^ with a possible diagnosis of ACS, but multiple studies have demonstrated its initial low sensitivity (28–65%) in diagnosing AMI.^7^, ^8^, ^9^, ^10^, ^11^ The use of cardiac serum markers as a supplement to the ECG has become standard in the assessment and risk stratification of acute myocardial ischemic injury.^12^, ^13^, ^14^, ^15^, ^16^ In fact, serum troponin evaluation has recently become a gold standard for the diagnosis of myocardial necrosis.^17^, ^18^, ^19^, ^20^ However, serum troponin results are generally not immediately available, such that the emergency physician (EP) must implement AMI treatment protocols by relying only on the initial patient evaluation and associated 12‐lead ECG interpretation.

The importance of quickly obtaining a measured 12‐lead ECG (mECG) cannot be underestimated in patients with a presentation suggestive of ACS. It is well known that the cardiac electrical field is based on a dipolar hypothesis.^21^ In theory, only three lead vectors should be needed to describe the dipolar cardiac electrical field. This concept was validated by Schreck^22^, ^23^ using nonlinear mathematical modeling demonstrating the accurate computerized derivation of the 12‐lead ECG from just three leads using a universal patient transformation matrix (UPTM). Since standard cardiac rhythm monitors can acquire and display three leads, it now becomes possible to quickly derive and display the 12‐lead ECG instantaneously directly from a cardiac rhythm monitor.

This study reports on the validation of a nonlinear mathematical model and the diagnostic performance of a new continuous cardiac “electrical” biomarker (CEB) for the detection of ECG changes suggestive of acute myocardial ischemic injury including AMI. This CEB is constructed directly from a derived 12‐lead ECG (dECG) that is UPTM‐synthesized from just three leads using only five body surface electrodes connected to a bedside cardiac monitor. The goal of this study was to identify and measure the diagnostic accuracy of this new CEB.

## METHODS

This study is an observational, retrospective, case‐control, blinded, noninferiority design comparing the diagnostic accuracy of a new CEB diagnostic test (Vectraplex ECG System with Vectraplex AMI, VectraCor, Inc., Totowa, NJ) against three active controls (AC) using two blinded physicians (board‐certified emergency medicine specialist and board‐certified cardiologist) as the reference standards for the 12‐lead ECG interpretations suggestive of acute myocardial ischemic injury including AMI. All ECG records were retrospectively reviewed. The institutional review board approved the study methodology and exempted the need for informed consent.

The mECGs from both AMI and non‐AMI patients were obtained from three established patient ECG databases obtained from the Physiobank^24^ archive including (1) the Physikalisch‐Technische Bundesanstalt (PTB) database; (2) the St.‐Petersburg Institute of Cardiological Technics 12‐lead Arrhythmia Database; and (3) the OpenECG^25^ database.

A fourth database from Muhlenberg Regional Medical Center (MRMC, Plainfield, NJ) was also used. This database includes consecutive patients that were admitted to the ED with chest pain. Patients included men and women, age ≥18. The standard mECGs were acquired using a Marquette MAC‐15 machine (GE Healthcare, Waukesha, WI).

**Figure 1:**
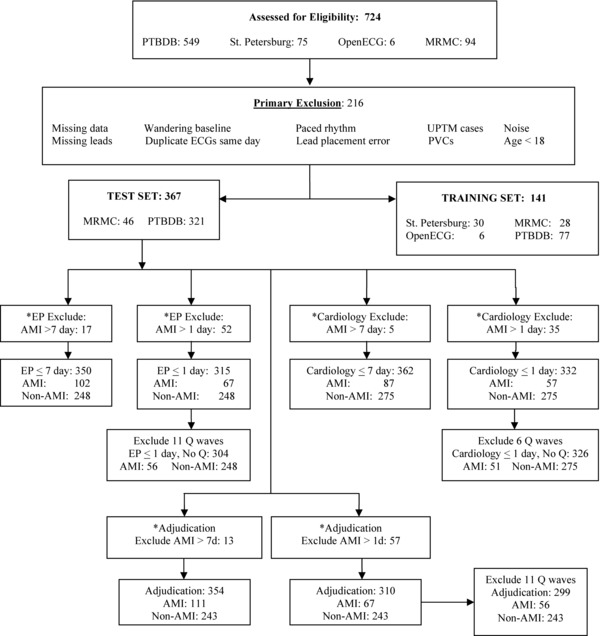
Flow diagram for case enrollment. *Note: Differences between EP, adjudication, and cardiology sample sizes are due to diagnosis and associated exclusion criteria. ECG = electrocardiogram; UPTM = universal patient transformation matrix; PVC = premature ventricular contraction; EP = emergency physician; AMI = acute myocardial infarction.

The Standards for the Reporting of Diagnostic accuracy studies (STARD)^26^ flow chart for case selection is shown in Figure 1. A total of 724 patients were assessed for eligibility. To minimize selection bias, all database cases were analyzed and subjected to the eligibility criteria. All consecutive database ECGs of both men and women were screened for inclusion. ECG cases were excluded from analysis for age <18, wandering baseline ≥5 mm, excessive noise, missing leads in the basis measured lead set, missing data, lead placement error, ventricular ectopy, duplicate ECGs acquired on the same calendar day, paced beats within the 10 seconds of captured ECG complexes, and those ECGs used to construct the UPTM. Consecutive mECG cases from all of the databases utilized were enrolled to minimize selection bias.

The physician reference standards were blinded to (1) the ECG acquisition and signal processing, (2) the CEB, (3) each other's ECG interpretations, and (4) to whether the 12‐lead ECG was measured or derived. Adjudication of the blinded results was performed by consensus with discrepant resolution analysis.

The ECG acquisition process used to derive the 12‐lead ECG has been previously described.^23^, ^27^ Briefly, the digitized voltage–time points are acquired for the eight measured ECG leads. Leads III, aVR, aVL, and aVF are calculated from known geometries in the Einthoven triangle^28^ and are redundant. The P–P full cycles in each 10 seconds of voltage–time data are averaged yielding a “median beat.”

The basis measured three‐lead set {I, II, and V2} was used to derive the remaining nine leads of the dECG using a UPTM that was constructed using a nonlinear optimization technique.^29^ Abstract factor analysis^30^ was performed to calculate the eigenvectors of the normalized, calibrated dECG voltage–time data in order to construct the CEB. For convenience, the mathematical formulation for this process is shown in the Appendix. Using this process, the primary eigenvectors of each voltage–time ECG data array can be identified for both the 12‐lead mECG and dECG. The CEB is constructed from multiple cycles in the digitized electrical data in the basis measured three‐lead set using a computerized analysis of the nonlinear mathematical transformations yielding the dECG^27^ and the associated eigenvectors representing the energy activity contributions in the dECG. The eigenvalues corresponding to the independent eigenvectors 3–8 were found to be highly significant (P < 0.01) for the mECG and dECG. However, the purpose of the VectraplexECG device is to derive the 12‐lead ECG from just three measured leads. Since the dECG has only three independent eigenvectors, the CEB is essentially a quantification of the dipolar activity in the cardiac electrical field.

The CEB is displayed as a numerical index on the cardiac monitoring device yielding a probability severity assessment of acute coronary obstructive disease leading to ACS. The CEB numerical index reflects the dipolar versus multipolar forces in the cardiac electrical field in order to distinguish the presence or absence of acute myocardial ischemic injury including AMI. The CEB is constructed from the dECG^27^ directly from the cardiac rhythm monitoring device (Vectraplex ECG System with Vectraplex AMI) using only three leads (five body surface electrodes).

This study was designed to simulate real‐world emergency medicine practice in which cardiac serum markers such as troponin are not immediately available to the EP. As such, the blinded physician interpretations of the mECGs and dECGs were used as the reference standards for the presence or absence of an ACS (acute myocardial ischemic injury including AMI). The criteria used for ECG changes suggestive of AMI have been previously defined.^31^, ^32^

A training set of 141 ECGs was established with 33 AMI and 108 non‐AMI cases from all the cases in the St. Petersburg database. In order to achieve sample size estimates and to broaden the population base of data, consecutive cases in portions of the Open ECG, PTB, and MRMC databases were also accessed. The purpose of the training set is to identify crude estimates of receiver‐operating‐characteristic (ROC) cutoffs for CEB diagnostic performance in an early phase trial.^33^ All remaining 367 consecutive ECG cases in the PTB and MRMC databases were included in the test set according to inclusion and exclusion criteria. ECGs acquired within 7 days from initial event were interpreted by the blinded physician reference standards. The 7‐day acute myocardial ischemic injury/AMI cutoff for ECG study inclusion was used to be consistent with the definition of “acute” myocardial ischemic injury that was generally accepted at the time of this investigation as proposed by Thygesen^31^ in 2007, which stated that AMI is temporally classified as “acute” when occurring in the “6 hours to 7 days” time frame. Thygesen updated this definition^32^ in August 2012 but it was not available at the time of this study.

A subanalysis of ECGs acquired within 1 day from initial event, indicative of a more acute presentation, was also performed and then stratified by the absence of significant Q waves. This was done to distinguish ECGs that contained Q‐wave necrosis patterns from ECGs with acute myocardial ischemic injury (acute ST–T wave changes alone) not containing significant Q‐wave necrosis patterns.

**Figure 2:**
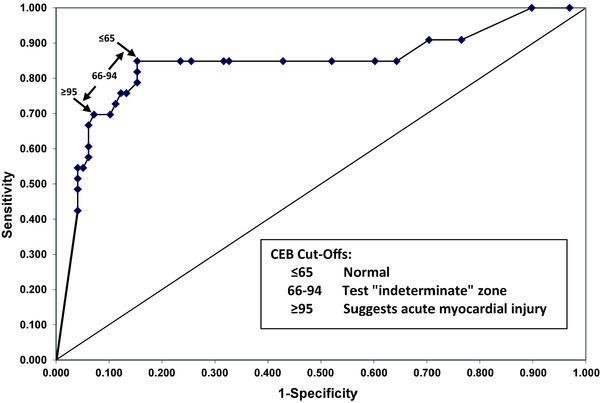
ROC analysis CEB training set. CEB = cardiac electrical biomarker.

The CEB diagnostic performance ROC cutoffs were identified from the training set ROC curve. In order to minimize spectrum bias, a CEB “indeterminate zone” was identified from the ROC curve. This approach is consistent with the interpretation scheme used for cardiac serum markers such as troponin. The ROC indeterminate zone was less than 10% of cases yielding greater than 90% test utility. This resulted in a cutoff point below which the CEB is considered negative for myocardial injury (<66), a small cutoff indeterminate range (66–94), and a cut‐off above which the CEB detects ECG changes suggestive of acute myocardial injury including AMI (>94). The training set ROC curve for CEB analysis is shown in Figure 2.

The CEB was compared against each AC including ST segment voltage analyses at different time (ms) points: the J point (ST0) and the area under the ST segment curve (STSUM) at 0, 20, 60, and 80 ms after the J point. The CEB was also tested against a 12‐lead ECG computerized diagnostic interpretation (ECGI) algorithm (Cardionics SA, Brussels, Belgium) as an additional AC.

Statistical design, confidence interval (CI) analysis, and sample size estimates of this study were based on the CEB:AC ratio^34^, ^35^ of the observed diagnostic performance parameters as analyzed in a paired noninferiority 1‐tail design with an α error of 0.025 and powered for 1‐β error of 0.9. Sensitivity, specificity, negative and positive predictive values were calculated to assess CEB versus AC diagnostic performance.

CIs and formulas were constructed based on a literature review^7^, ^8^, ^9^, ^10^, ^11^ using a 14% noninferiority margin for sensitivity (corresponding to CEB:AC margin ratio^34^, ^35^ of 0.8) and 7.5% for specificity (corresponding to a CEB:AC margin ratio^34^, ^35^ of 1.75).

The actual CEB diagnostic accuracy parameters were calculated based on the exclusion of the “indeterminate zone” cases. However, the worst case scenarios were also calculated by including all indeterminate CEBs as either a false positive or false negative result.

## RESULTS

The 141 training set cases had a median age of 58.5 ± 13.1 years and included 55.3% men with a median age of 59.1 ± 12.4 years. The remaining training set women had a median age of 57.0 ± 12.4 years. There was a 22.7% overall prevalence of AMI in the training set. The characteristics of the test set cases are shown in Table 1. Sample sizes of these sets met the estimated requirements.^34^, ^35^ ECGs acquired within 7 days from initial event were studied to be consistent with the reported definition of AMI.^31^ However, this definition could be considered to be based largely on pathological changes. Many clinicians consider the acute presentation of myocardial ischemia to occur on the day of presentation. As such, a subanalysis was performed on ECGs acquired ≤1 day from initial event.

**Table 1:** Characteristics of the Test Set Cases

	ECGs ≤ 7 Days	ECGs ≤ 1 Day
n	367	310
Male (%)	71.4	70.3
Age (all)	55.8 ± 14.4	54.8 ± 14.7
Median age (male)	54.2 ± 13.5	54.0 ± 13.5
Median age (female)	59.8 ± 15.8	58.6 ± 16.2
Non‐AMI (%)	66.2	78.4
Median age non‐AMI	52.4 ± 14.4	52.4 ± 14.4
Male % non‐AMI	75.3%	71.6%
Median age male non‐AMI	51.7 ± 13.9	51.7 ± 13.9
Median age female non‐AMI	54.1 ± 15.5	54.1 ± 15.5
AMI (%)	33.8	21.6
Median age AMI	62.4 ± 11.8	63.8 ± 12.0
Male AMI (%)	71.0	65.7
Median age male AMI	59.2 ± 11.0	59.7 ± 10.8
Median age female AMI	70.3 ± 10.1	71.6 ± 10.2
STEMI (all) (%)	67.7	79.1
NSTEMI (all) (%)	32.3	20.9
% AMI without Q wave	66.1	62.7
Inferior wall AMI (%)	37.1	49.3
Lateral wall AMI (%)	16.9	22.4
Anterior wall AMI (%)	30.6	29.9
Septal wall AMI (%)	27.4	26.9
Posterior wall AMI (%)	16.9	25.4

STEMI = ST elevation myocardial infarction; NSTEMI = Non‐ST elevation myocardial infarction.

Other abbreviations as in text.

**Figure 3:**
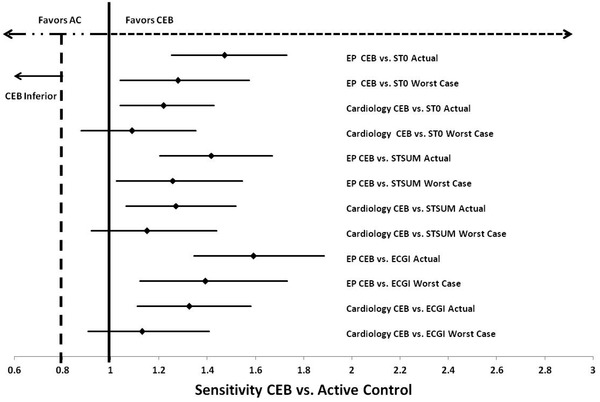
Sensitivity CEB versus AC (ST0, STSUM, ECGI) with 95% CI in 12‐lead ECGs acquired ≤7 days after initial presentation interpreted by EP and cardiologist. ECG = electrocardiogram; AC = active control; CEB = cardiac electrical biomarker; ST0 = J point; EP = emergency physician; STSUM = ST segment area under curve; ECGI = ECG computer interpretation.

**Figure 4:**
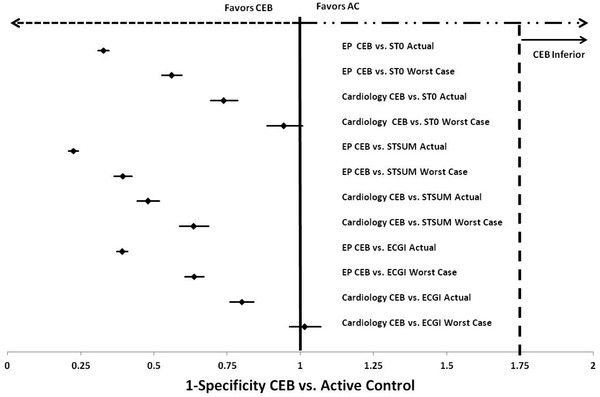
One‐specificity CEB versus AC (ST0, STSUM, ECGI) with 95% CI in 12‐lead ECGs acquired ≤7 day after initial presentation interpreted by EP and cardiologist. ECG = electrocardiogram; AC = active control; CEB = cardiac electrical biomarker; ST0 = J point; EP = emergency physician; STSUM = ST segment area under curve; ECGI = ECG computer interpretation.

The CEB diagnostic performance summary from ECGs acquired ≤7 days from initial event as interpreted by the two independent blinded physician reference standards, compared against the ACs, is shown in Table 2 with the adjudication results. In all analyses the CEB demonstrated noninferiority to ACs by hypothesis testing. The CEB sensitivities and specificities were significantly higher (P < 0.025) in the cardiologist STSUM and ECGI analyses, and significantly higher in all three comparative AC analyses by the EP and adjudication. High CEB negative predictive values were demonstrated in all analyses and CEB positive predictive values were improved over the ACs. The adjudicated likelihood ratios of a positive and negative CEB test were 12.2 and 0.158, respectively. The corresponding CEB sensitivity and 1‐specificity with 95% CI in the paired analyses,^34^, ^35^ respectively, against ACs are shown in Figures 3 and 4. CEB noninferiority was demonstrated to all ACs in each analysis. It is important to note that noninferiority was also demonstrated in all worst case scenarios. However, although this study is a noninferiority design, the actual data CEB performance showed superiority to the ACs according to the 95% CI analyses for both sensitivity and specificity in all actual data comparisons.

**Table 2:** CEB Diagnostic Performance in ECGs Acquired ≤ 7 Day from AMI Event

	CEB vs ST0			CEB vs STSUM			CEB vs ECGI		
Diagnostic Parameter	EP	Cardiology	Adjudicate	EP	Cardiology	Adjudicate	EP	Cardiology	Adjudicate
Sensitivity CEB (%)	87.6	80.3	85.3	87.6	80.3	85.3	87.6	80.3	85.3
Sensitivity AC (%)	59.6	65.8	54.7	61.8	63.2	56.8	55.1	60.5	58.9
Specificity CEB (%)	91.3	81.2	93.0	91.3	81.2	93.0	91.3	81.2	93.0
Specificity AC (%)	73.6	74.5	72.2	61.5	60.8	76.6	77.9	76.5	80.6
NPV CEB (%)	95.0	93.2	93.8	95.0	93.2	93.8	95.0	93.2	93.8
NPV AC (%)	82.5	88.0	79.2	80.7	84.7	76.6	81.8	86.7	82.4
PPV CEB (%)	79.6	56.0	83.5	79.6	56.0	83.5	79.6	56.0	83.5
PPV AC (%)	46.5	43.5	45.2	38.2	32.4	36.7	49.0	43.4	56.0
Prevalence (%)	27.8	23.0	29.5	27.8	23.0	29.5	27.8	23.0	29.5

STO = J point; NPV = negative predictive value; PPV= positive predictive value.

Other abbreviations as in text.

**Figure 5:**
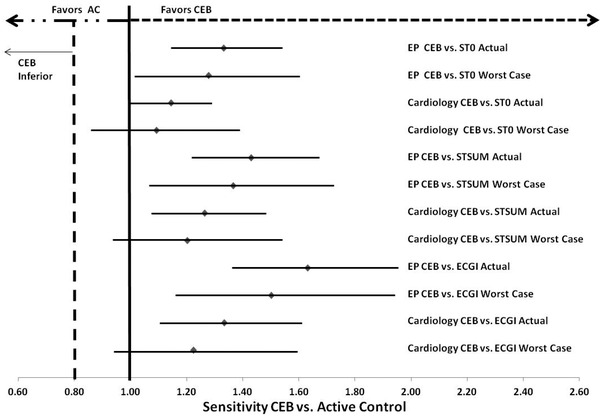
Sensitivity CEB versus AC (ST0, STSUM, ECGI) with 95% CI in 12‐lead ECGs, with development of significant Q waves included, acquired ≤1 day after initial presentation interpreted by EP and cardiologist. ECG = electrocardiogram; AC = active control; CEB = cardiac electrical biomarker; ST0 = J point; EP = emergency physician; STSUM = ST segment area under curve; ECGI = ECG computer interpretation.

**Figure 6:**
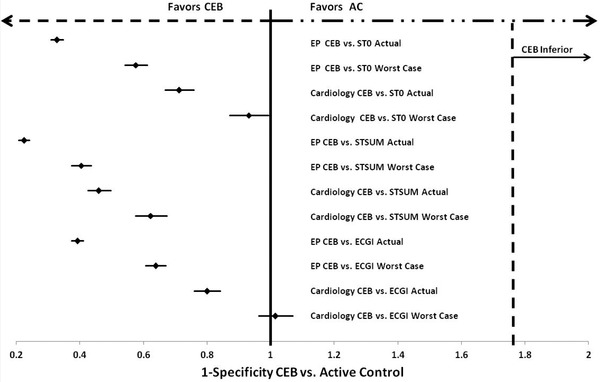
One‐specificity CEB versus AC (ST0, STSUM, ECGI) with 95% CI in 12‐lead ECGs, with development of significant Q waves included, acquired ≤1 day after initial presentation interpreted by EP and cardiologist. ECG = electrocardiogram; AC = active control; CEB = cardiac electrical biomarker; ST0 = J point; EP = emergency physician; STSUM = ST segment area under curve; ECGI = ECG computer interpretation.

The CEB diagnostic performance summary from ECGs acquired ≤1 day from initial event as interpreted by the two independent blinded physician reference standards, compared against the ACs, is shown in Table 3 with the adjudication results. Again, the CEB sensitivities and specificities were significantly higher (P < 0.025) in the cardiologist STSUM and ECGI analyses, and significantly higher in all three comparative AC analyses by the EP and adjudication. The adjudicated likelihood ratios of a positive and negative CEB test were 13.1 and 0.088, respectively. Figures 5 and 6 show the sensitivity and 1‐specificity analyses, respectively, for paired design analysis for all test set ECGs and demonstrate CEB noninferiority to all ACs in each analysis. Noninferiority was also demonstrated in all worst case scenarios. The actual data CEB performance again shows superiority to the ACs according to the CI analyses for both sensitivity and specificity.

**Table 3:** CEB Diagnostic Performance in ECGs Acquired ≤ 1 Day from AMI Event

	CEB vs ST0			CEB vs STSUM			CEB vs ECGI		
Diagnostic Parameter	EP	Cardiology	Adjudicate	EP	Cardiology	Adjudicate	EP	Cardiology	Adjudicate
Sensitivity CEB (%)	93.8	87.3	91.8	93.8	87.3	92.0	93.4	84.6	91.8
Sensitivity AC (%)	70.3	76.4	65.6	65.6	69.1	61.0	57.4	63.5	59.0
Specificity CEB (%)	91.3	81.5	93.0	91.3	81.8	93.0	91.3	81.2	93.0
Specificity AC (%)	73.5	74.0	72.2	61.3	60.5	59.0	77.9	76.5	80.6
NPV CEB (%)	98.1	96.7	97.7	98.1	96.7	97.7	98.1	96.3	97.7
NPV AC (%)	89.9	93.5	88.6	86.5	90.0	84.8	87.4	91.1	88.0
PPV CEB (%)	75.0	50.5	77.8	75.0	51.1	77.8	74.0	47.8	77.8
PPV AC (%)	42.5	38.9	38.8	32.1	27.5	28.5	40.7	35.5	45.0
Prevalence (%)	19.0	17.4	21.2	21.8	17.9	21.2	20.9	16.9	21.2

STO = J point; NPV = negative predictive value; PPV= positive predictive value.

Other abbreviations as in text.

**Figure 7:**
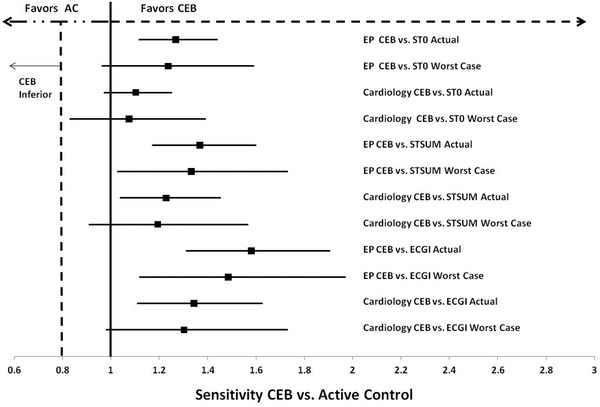
Sensitivity CEB versus AC (ST0, STSUM, ECGI) with 95% CI in 12‐lead ECGs, with development of significant Q waves excluded, acquired ≤1 day after initial presentation interpreted by EP and cardiologist. ECG = electrocardiogram; AC = active control; CEB = cardiac electrical biomarker; ST0 = J point; EP = emergency physician; STSUM = ST segment area under curve; ECGI = ECG computer interpretation.

**Figure 8:**
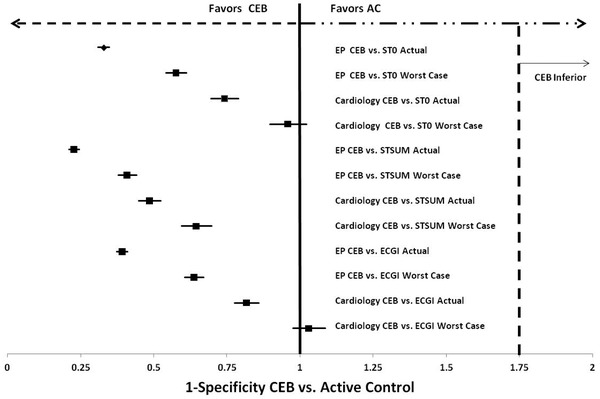
One‐specificity CEB versus AC (ST0, STSUM, ECGI) with 95% CI in 12‐lead ECGs, with development of significant Q waves excluded, acquired ≤1 day after initial presentation interpreted by EP and cardiologist. ECG = electrocardiogram; AC = active control; CEB = cardiac electrical biomarker; ST0 = J point; EP = emergency physician; STSUM = ST segment area under curve; ECGI = ECG computer interpretation.

Since Q‐wave development in the setting of acute ST changes can be an indicator of actual AMI, a subanalysis of ECGs without significant Q waves acquired 1 day from initial event was also performed. These cases are considered to represent actual acute myocardial ischemic injury before the progression to myocardial necrosis occurs. The CEB diagnostic performance of these ECG cases is shown in Table 4. Again, the CEB sensitivities and specificities were significantly higher (P < 0.025) in the cardiologist STSUM and ECGI analyses, and significantly higher in all three comparative AC analyses by the EP and adjudication. The adjudicated likelihood ratios of a positive and negative CEB test were 13.3 and 0.060, respectively. Figures 7 and 8 show the sensitivity and 1‐specificity paired analyses, respectively, and demonstrate CEB noninferiority to all ACs in each analysis. This analysis was performed on 12‐lead ECGs ≤ 1 day from initial event demonstrating acute myocardial ischemic injury prior to the development of significant Q waves consistent with concomitant myocardial necrosis. Noninferiority was also demonstrated in all worst case scenarios. Figures 7 and 8 also demonstrate actual data CEB sensitivity and specificity superiority to all ACs by the EP reference standard and CEB superiority to STSUM and ECGI by the cardiology reference standard as shown by the CI analysis.

**Table 4:** CEB Diagnostic Performance in ECGs (no Q Waves) Acquired ≤ 1 Day from AMI Event

	CEB vs ST0			CEB vs STSUM			CEB vs ECGI		
Diagnostic Parameter	EP	Cardiology	Adjudicate	EP	Cardiology	Adjudicate	EP	Cardiology	Adjudicate
Sensitivity CEB (%)	96.3	87.8	94.4	96.3	87.8	94.4	96.1	87.8	94.3
Sensitivity AC (%)	75.9	79.6	72.2	70.4	71.4	66.7	60.8	65.3	60.4
Specificity CEB (%)	91.3	80.8	92.9	91.3	80.8	92.9	91.3	80.8	92.5
Specificity AC (%)	73.5	74.1	72.1	61.7	60.4	57.5	77.9	76.5	80.2
NPV CEB (%)	99.1	97.2	98.6	99.1	97.2	98.6	99.1	97.2	98.6
NPV AC (%)	92.9	95.0	91.6	89.9	91.7	87.8	90.0	92.0	89.7
PPV CEB (%)	72.2	46.7	76.1	72.2	46.7	76.1	71.0	46.7	74.6
PPV AC (%)	40.2	37.1	38.2	30.2	25.7	23.3	37.8	34.8	41.6
Prevalence (%)	19.0	16.1	19.3	19.0	16.1	19.3	18.1	16.1	18.9

STO = J point; NPV = negative predictive value; PPV= positive predictive value.

Other abbreviations as in text.

The percent agreements^36^, ^37^ between the dECG and mECG are shown in Table 5. There is strong intra‐ and interagreement between the mECG and dECG for acute myocardial ischemic injury by both blinded reference standards.

**Table 5:** Reference Standard 12‐Lead ECG Interpretation of Acute Myocardial Ischemic; Injury Including AMI: Inter‐ and Intra‐Agreement of dECG versus mECG

12‐Lead ECG AMI Interpretation	Overall	Positive	Negative
Reference Standard Category	Agreement % (95% CI)	Agreement % (95% CI)	Agreement % (95% CI)
EP: mECG vs dECG	100 (99.1, 99.9)	100 (98.9, 100)	100 (98.5, 100)
Card: mECG vs dECG	98.9 (97.6, 99.2)	96.7 (90.8, 98.9)	98.9 (98.0, 99.9)
EP vs Card: mECG	92.6 (90.3, 94.1)	93.1 (85.8, 96.8)	92.5 (88.8, 95.0)
EP vs Card: dECG	92.9 (90.6, 94.4)	95.2 (88.4, 98.1)	92.2 (88.5, 94.8)

Card = cardiologist.

Other abbreviations as in text.

## DISCUSSION

Rapid diagnosis of acute myocardial ischemic injury is the key to implementing immediate treatment. Although serum cardiac markers, especially troponin, are now routine, their sensitivities vary and are highly dependent on time from onset of symptoms^38^ making serial measurement a necessity during patient observation in the ED.^39^ For presumed ACS patients, it is usual and customary to acquire serial 12‐lead ECGs and cardiac serum markers at the time of patient arrival, and every several hours thereafter, for up to 24 hours of patient observation to identify the development of an ACS. The patient may be at risk during the time between these serum markers and ECG acquisitions, especially if the patient has silent ischemic episodes.

Basic cardiac monitoring technology only enables the display of heart rate and rhythm. Continuous 12‐lead ECG ST‐segment monitoring was developed to increase the detection of acute myocardial ischemic events and has been reported to bring clinical advantages^40^ but is underused due to a variety of issues^41^ including false positive alarms, lack of hardware or software for accurate ST‐segment analysis, accurate ECG signal transmission to the ST monitor, acceptance of practice standards and guidelines, and a lack of consensus by physicians about the necessity of continuous ST‐segment monitoring. Also, it is recognized that connecting a dedicated 12‐lead ECG machine in addition to a cardiac monitor for every ED patient with chest pain is not feasible.

It would be greatly advantageous in acute care settings to use the cardiac rhythm monitor to detect continuously the development of acute myocardial ischemic injury in patients at risk for ACS using a noninvasive CEB. This occurs continuously, especially in between the times for serial 12‐lead ECG and cardiac serum marker acquisitions when patients are at greatest risk while being observed. The rapid diagnosis of acute myocardial ischemic injury using a CEB obtained and displayed continuously on the cardiac rhythm monitor could lead to more rapid therapeutic interventions and hopefully decrease myocardial injury progression and lessen the resulting morbidities such as arrhythmia and congestive heart failure. This strategy could also help limit repeat hospital admissions by limiting such morbidities.

This study was performed to identify and test a new CEB that is obtained directly from the cardiac monitor in patients at risk for the development of acute myocardial ischemic injury including AMI. This is the first study to recognize the concept of the CEB, as distinguished from cardiac “serum” biomarkers, in that the CEB can be observed continuously and noninvasively using this currently available ECG and cardiac monitoring device. This CEB can be an advantageous adjunct to 12‐lead ECG and cardiac serum biomarker determination when evaluating patients with a potential ACS. The reported high negative predictive value results are an advantage to clinicians practicing in acute care areas including the ED.

The CEB behavior involves understanding of the concepts of dipolar and multipolar activity contributions to the cardiac electrical field.^21^, ^42^, ^43^, ^44^ Although it is commonly taught that the cardiac electrical field is “dipolar,”^21^ there are very small but measurable multipolar contributions that exist. Multipolar contributions^42^ to the cardiac electrical field may be associated with pathology such as acute infarction.^42^, ^45^, ^46^ This study demonstrates a correlation between the CEB and acute myocardial ischemic injury by quantifying the very small but measurable multipolar contributions to the electrical field. It appears from the results that the more multipolar activity in the cardiac electrical field, the more likely is the presence of acute myocardial ischemic injury.

The diagnostic performance of the ACs in this study was shown to be consistent with that reported in the literature. The diagnostic performance of the CEB is shown to be noninferior by the a priori hypothesis testing design. It is interesting that the EP analysis showed a higher diagnostic performance than the cardiology analysis. This is not unexpected since EPs may tend to over read the 12‐lead ECG in clinical practice to decrease the false negative rate. Discrepant resolution by consensus adjudication was performed to mitigate this limitation.

As shown in Table 2, in the ≤7‐day AMI ECG analysis, the CEB performed better than each of the ACs. ECGs obtained ≤1 day from initial presentation revealed a slightly better sensitivity. These cases were considered to be the highest measure of “acute” ischemic injury. It is possible that the cardiac electrical field reorganizes over time after an acute injury, but investigations of the use of the CEB as a prognostic risk stratification marker are the subject of future prospective studies. The CEB sensitivity of approximately 90%, while maintaining a specificity of approximately 90%, is quite striking given that this marker is obtained instantly and noninvasively on a cardiac rhythm monitor. Given the excellent CEB test likelihood ratios in the reference standard and adjudication analyses, this is a marked improvement over conventional cardiac monitoring and 12‐lead ECG acquisition.

The main limitation of this study is its retrospective design. Selection bias was addressed by screening all cases in the ECG databases for inclusion in the study.

Another limitation is that the reference standard used in this study is imperfect. Each physician expert reader has their own inherent error in the interpretation of each 12‐lead ECG. The rationale for the reference standard design was to simulate the real‐world ED practice in that the EP is often the first physician to interpret the ECG for the presence of AMI, and then contact the cardiologist with the initial ECG interpretation. Emergency therapy can then be implemented, even though cardiac serum biomarkers may not yet be available. However, the interpretation of the initial 12‐lead ECG in the ED setting by the EP and/or the cardiologist is still a standard of care in the assessment of cardiac disease. Adjudication discrepant analysis was performed to mitigate this limitation.

There was no direct comparison with cardiac serum biomarkers since markers such as troponin are generally not immediately available to assist the EP in activating protocols for emergency care. These limitations were understood and accepted a priori given that the diagnosis of acute myocardial injury in the ED for the purpose of implementing immediate therapy (i.e., fibrinolytic administration or transport to a cardiac catheterization laboratory for intervention) is based only on the patient clinical presentation and 12‐lead ECG interpretation.

Sample size limitations did not allow the subanalyses of race, age, and severity of disease considerations. Also, the use of the CEB as a prognosticator, such as has been reported for troponins,^47^ cannot be considered at this time due to the retrospective data analysis. Future prospective studies in this regard are necessary.

An analysis of gender was not included in this study since the UPTM was designed to be all inclusive and independent of gender, race, age, timing, and body habitus.

A further limitation may be the definition of AMI itself. In this study, acute cardiac ischemic injury included those cases with acute ECG ST segment and T‐wave changes with or without Q‐wave development. Infarction was considered to have occurred when significant Q waves were present and was considered “acute” when accompanied by the accepted standards of ST segment or T‐wave ischemic injury abnormalities.

In summary, this study demonstrates that a new CEB has been identified that detects ECG changes suggestive of acute myocardial ischemic injury including AMI with high diagnostic accuracy. This CEB is instantly constructed directly from the cardiac rhythm monitor and displayed continuously using only five body surface monitoring electrodes (three lead vectors). Additionally, it has been demonstrated that the standard 12‐lead ECG can be derived with accuracy from these same three leads using just the cardiac monitor. This process occurs continuously while monitoring patients at risk for ACS. The CEB has higher diagnostic accuracy than the customary ST voltage analyses of the standard 12‐lead ECG and the associated computerized ECG interpretation algorithm. This process allows an immediate, cost‐effective, and efficient means of identifying patients with acute myocardial ischemic injury including AMI who are being monitored in the ED and other acute care settings.

## Prior Presentations

The Synthesis of Normal and Infarction Electrocardiograms from Principal Vectors by Factor Analysis. Schreck, DM et al. Presented at the Fourth International Conference on Emergency Medicine, May 9, 1992, Washington, D.C.

Mathematical Modeling in Electrocardiography (educational exhibit). Schreck, DM et al. Presented at the American College of Emergency Physicians Scientific Assembly September 14–16, 1992, Seattle, Washington.

Factor Analysis of the Standard Electrocardiogram. Schreck DM. Presented at the Society for Academic Emergency Medicine Annual Scientific Sessions, May 7, 1996, Denver, Colorado.

On the Derivation of a New Cardiac Marker: The Electrocardiogram Eigenvalues. Schreck DM, Brotea C, Shah SP. Presented at the American College of Emergency Physicians Annual Scientific Sessions, October 8, 2002, Seattle, Washington.

A New Marker for Myocardial Infarction: A Real‐Time Computer Model for Measured and Derived ECG Eigenvalues. Schreck DM. Presented at the Society of Critical Care Medicine 32nd Critical Care Congress, February 1, 2003, San Antonio, Texas.

A Real‐Time Cardiac Marker for Acute Infarction: Eigenvalues of the Measured and Derived ECG. Schreck DM. American College of Emergency Physicians Annual Research Forum. October 12, 2003, Boston, Massachusetts.

Clinical Application of Eigenvalue Analysis and the Derived Electrocardiogram as a Real‐Time Marker for Infarction. Schreck DM. Presented at the American College of Cardiology Annual Scientific Sessions 2004, March 9, 2004, New Orleans, Louisiana.

The Eigenvalue as an Electrical Marker in the Prediction of Acute Myocardial Infarction in Measured and Derived Electrocardiograms. Schreck, DM. Presented at the Computers in Cardiology Annual Scientific Sessions, September 21, 2004, Chicago, Illinois.

The Eigenvalue Model as an Electrical Marker in the Prediction of Acute Myocardial Infarction in Measured and Derived Electrocardiograms. Schreck, DM. Presented at the American College of Emergency Physicians Research Forum, Ocotber 18, 2004, San Francisco, California.

The Eigenvalue Model as an Electrical Marker in the Prediction of Acute Myocardial Infarction in Measured and Derived Electrocardiograms. Schreck, DM. Presented at the American Heart Association Annual Scientific Sessions, November 10, 2004, New Orleans, Louisiana.

The Derived ECG Eigenvalues: A Marker for Acute Infarction. Schreck DM. Presented at the International Society for Computerized Electrocardiology, April 15, 2005, Kauai, Hawaii.

The Eigenvalues of the Electrocardiogram: A New Electrical Cardiac Marker for Acute Myocardial Infarction. Schreck DM. Presented at the American College of Chest Physicians, November 2, 2005. Montreal, Canada.

The Derived Electrocardiogram Eigenvalues as a Marker for Acute Myocardial Infarction. Schreck DM, et al. Presented at the Society for Academic Emergency Medicine Annual Meeting. May 17, 2007, Chicago, Illinois.

Eigenvalue Analysis of the 15‐Lead Electrocardiogram. Schreck DM, et al. Presented at the 4th Mediterranean Emergency Medicine Conference. September 17, 2007, Sorrento, Italy.

Eigenvalue Analysis of the 15‐Lead Electrocardiogram. Schreck DM. Presented at the Society for Critical Care Medicine 37th Critical Care Congress. February 3, 2008. Honolulu, Hawaii.

The Derived Electrocardiogram and a Cardiac Electrical Marker for Acute Myocardial Infarction. Schreck DM. Presented at the American College of Emergency Physicians Research Forum. October 28, 2008. Chicago, Illinois.

Eigenvalue Analysis as an Electrical Cardiac Biomarker to Identify Acute Myocardial Infarction.

Schreck DM. Presented at the American College of Emergency Physicians Research Forum. September 28, 2010. Las Vegas, Nevada.

Detection of Acute Myocardial Infarction Using a Real‐Time Cardiac Electrical Biomarker. Schreck DM. Presented at the Society of Critical Care Medicine 40th Annual Critical Care Congress. January 16, 2011. San Diego, California.

Real‐Time Cardiac Electrical Biomarker for Detection of AMI. Schreck DM and Fishberg RD. Presented at the American College of Cardiology Annual Scientific Sessions. April 16, 2011. New Orleans, Louisiana.

Real‐Time Cardiac Electrical Biomarker for Detection of Acute Myocardial Ischemic Injury. Schreck DM and Fishberg RD. Presented at the Society of Critical Care Medicine 42d Annual Critical Care Congress. January 20, 2013. San Juan, Puerto Rico.

## Mathematical Formulation of Abstract Factor Analysis

Let D represent a data matrix array. A covariance matrix can then be constructed by multiplying D by its transpose as follows:

(A1) $${Z=D}^{T}\;D,$$

where Z is the covariance matrix.

The covariance matrix Z is then diagonalized by finding a matrix Q such that

(A2) Q−1ZQ=[λjδ jk ],

where δ_jk_ is the Kronecker delta such that,

(A3) δ jk =0 if j≠k,δ jk =1 if j=k,

and λ_j_ is an eigenvalue of the set of equations

(A4) $$Z\;q_{j}\;=\;\lambda_{j}\;q_{j},$$

and where q_j_ is the j‐th column of Q eigenvectors.
